# Lipopolysaccharide derived from the rumen down-regulates stearoyl-CoA desaturase 1 expression and alters fatty acid composition in the liver of dairy cows fed a high-concentrate diet

**DOI:** 10.1186/s12917-015-0360-6

**Published:** 2015-03-07

**Authors:** Tianle Xu, Hui Tao, Guangjun Chang, Kai Zhang, Lei Xu, Xiangzhen Shen

**Affiliations:** College of Veterinary Medicine, Nanjing Agricultural University, Nanjing, 210095 China

**Keywords:** Lipopolysaccharide, Stearoyl-CoA desaturase 1, Long chain fatty acid, Liver, High concentrate diet, Dairy cows

## Abstract

**Background:**

Dairy cows are often fed a high-concentrate diet to meet lactating demands, yet long-term concentrate feeding induces subacute ruminal acidosis (SARA) and leads to a decrease in milk fat. Stearoyl-CoA desaturase1 (SCD1) participates in fatty acid biosynthesis in the liver of lactating ruminants. Here, we conducted this study to investigate the impact of lipopolysaccharide derived from the rumen on SCD1 expression and on fatty acid composition in the liver of dairy cows fed a high-concentrate diet. Eight multiparous mid-lactating Holstein cows (455 ± 28 kg) were randomly assigned into two groups in the experiment and were fed a low-concentrate diet (LC) or high-concentrate diet (HC) for 18 weeks.

**Results:**

The results showed that the total volatile fatty acids and lactic acid accumulated in the rumen, leading to a decreased rumen pH and elevated lipopolysaccharides (LPSs) in the HC group. The long chain fatty acid profile in the rumen and hepatic vein was remarkably altered in the animals fed the HC diet. The triglyceride (TG), non-esterified fatty acid (NEFA) and total cholesterol (TCH) content in the plasma was significantly decreased, whereas plasma glucose and insulin levels were increased. The expression of SCD1 in the liver was significantly down-regulated in the HC group. In regards to transcriptional regulators, the expression of sterol regulatory element binding transcription factors (SREBF1c, SREBF2) and SREBP cleavage activating protein (SCAP) was down-regulated, while peroxisome proliferator-activated receptor α (PPARα) was up-regulated.

**Conclusions:**

These data indicate that lipopolysaccharide derived from the rumen down-regulates stearoyl-CoA desaturase 1 expression and alters fatty acid composition in the liver of dairy cows fed a high-concentrate diet.

## Background

Dairy cows are often fed a high-concentrate diet to meet lactating requirements for high milk performance [1]. However, long-term feeding with a high-concentrate diet causes a decline in the rumen pH if organic acids, such as volatile fatty acids (VFAs) and lactic acid, accumulate in the rumen [2,3], and a chronic digestive disorder known as subacute ruminal acidosis (SARA) may occur. A rumen pH of less than 5.6 for over 3 h per day is used as a parameter to determine the occurrence of SARA [1]. Decreased rumen pH results in the release of lipopolysaccharides (LPSs), which originate from the cell-wall component of gram-negative bacteria [4].

Previous studies demonstrated that LPSs stimulate the gene expression of fatty acid synthetase (FAS) and acetyl-CoA carboxylase (ACC) in the liver of mice [5] but depressed stearoyl-CoA desaturase (SCD) expression in bovine hepatocytes [6]. SCD is a rate-limiting enzyme that catalyzes the synthesis of the monounsaturated fatty acids oleate (18:1) and palmitoleate (16:1) and forms triglycerides and cholesterol esters [7]. Microarray assays have indicated that the gene expression profile was altered in the liver of SCD knockout mice, and the most obvious pattern was down-regulation of the genes involved in lipogenesis and up-regulation of the genes associated with fatty acid β-oxidation [8]. It was reported that LPS modulates lipid metabolism by inhibiting the clearance of triacylglycerol in the livers of bovine [9]. Furthermore, in a LPS-induced liver injury model, SCD1 expression was inhibited in the liver of mice, suggesting the potential action of LPS on SCD1 inhibition [10].

The liver is responsible for lipid metabolism in ruminant animals, and SARA is associated with liver abscesses, fatty liver and a whole-body inflammatory response when a high-grain diet is offered [11]. Therefore, the repartition of energy from production to anti-inflammation may exist in the liver and consequently lead to a negative energy balance during long-term high-concentrate supply. Many studies have been carried out on milk fat depression (MFD) in diet-induced SARA [12]. Some studies have focused on the *trans* fatty acid (i.e., *trans-10* C18:1n) pathway [13], while others have paid attention to LPS, which initiates the inflammatory response and influences the fatty acid profile in the rumen and milk [14]. Currently, several studies have been performed to evaluate hepatic lipid metabolism in dairy cows via exogenous LPS infusion [15].However, less information is available in regards to the alterations in hepatic lipid metabolism during long-term diet-induced SARA in dairy cows. Therefore, the present study was conducted to investigate the effects of a high-concentrate diet on the fatty acid composition and SCD1 expression in the liver of dairy cows.

## Methods

### Animals, diets and experimental design

Eight multiparous mid-lactating Holstein cows (455 ± 28 kg) were randomly assigned into two groups. One group was fed with a high-concentrate diet (HC) composed of 40% forage and 60% concentrate as a treatment, and the other group was offered a low-concentrate diet (LC) composed of 60% forage and 40% concentrate as a control for the 18-week experimental period. The ingredients and nutritional composition of the diets are presented in Table 1. The cows were fitted with a rumen fistula and hepatic catheters two weeks before the experiment and were ensured that they recovered from the surgery. The animals were maintained in individual tie stalls, fed at 0400, 1200, and 2000 h, and had free access to fresh water throughout the experimental time period.

**Table 1:** **The ingredients in the diets and the nutritional composition**

**Ingredients, % of DM**	**LC** ^**1**^	**HC** ^**1**^
Corn silage	30	20
Alfalfa	30	20
Maize	22.78	33.6
Wheat bran	5.15	15
Soybean meal	9.81	9
Calcium phosphate dibasic	0.92	0.53
Powder	0	0.52
Salt	0.35	0.35
Premix^2^	1	1
Total	100	100
**Nutritional Composition** ^3^		
NE MJ/kg	6.32	6.74
CP %	16	16.2
EE %	3.96	4.15
NDF %	37.71	31.92
ADF %	22.75	17.55
NFC %	33.43	40.31
Ca %	0.9	0.8
P %	0.45	0.45

^1^ LC, low concentrate; HC, high concentrate.

^2^The premix contained VA,1,900ku/kg; VD, 250ku/kg; VE, 3,000 mg/kg; Niacin, 4,000 mg/kg; Cu, 1,200 mg/kg; Fe, 525 mg/kg; Zn, 13,000 mg/kg; Mn, 5,500 mg/kg; I, 170 mg/kg; Co, 50 mg/kg; Se, 27 mg/kg.

^3^The calculated nutritional composition values.

The animal experiment was reviewed and approved by the Institutional Animal Care and Use Committee of Nanjing Agricultural University. The experiment was performed in accordance with the “Guidelines for Experimental Animals” of the Ministry of Science and Technology (Beijing, China).

### Sample collection and analysis

The cows were milked at 0500, 1300, and 2100 h, and the milk yield was recorded daily. A 50-mL milk sample was taken to determine the milk fat and milk protein concentrations once a week (MilkoScan^TM^ FT1, FOSS, Denmark). Samples of the ruminal fluid were taken via the rumen fistula for 3 consecutive days during the 18^th^ week, at 2-h intervals starting at 0400 h (after the morning feeding) for 12 hours. The samples were filtered through 2 layers of cheesecloth and stored at −20°C for the LPS, VFA, lactic acid and long-chain fatty acid analyses. A blood sample was taken at the same time as the ruminal fluid collection via the hepatic vein catheter and from the jugular vein using 5-mL vacuum tubes containing sodium heparin as an anticoagulant. The plasma was isolated from the blood samples by centrifugation at 3000 × g at 4°C for 15 min and was stored at −20°C for the LPS, biochemical parameter, hormones and long chain fatty acid analyses. Liver tissue samples were taken using a punch biopsy with a local anesthesia, and the samples were frozen in liquid nitrogen and then stored at −70°C until the quantitative Real-Time PCR and western blotting analyses.

### LPS and biochemical parameters in the plasma and ruminal fluid

The LPS concentration in the ruminal fluid and plasma were determined using a chromogenic endpoint assay (CE64406, Chinese Horseshoe Crab Reagent Manufactory Co., Ltd., Xiamen, China) with a minimum detection limit of 0.05 EU/mL. The procedures were performed according to the manufacturer’s instructions.

The analyses for the triglyceride, NEFA, total cholesterol and glucose concentrations were performed using commercial kits (Glucose Assay Kit, Rongsheng, Shanghai, China; Nonesterified Free Fatty Acids Assay Kit, Jiancheng, Nanjing, China; Total Cholesterol Reagent Kit, Dongou, Zhejiang, China; Lactic Acid Assay Kit, Jiancheng, Nanjing, China; Triglyceride Reagent Kit, Jiancheng, Nanjing, China) that used an enzymatic colorimetric method read by a microplate reader (Epoch, BioTek, USA). Plasma insulin and glucagon concentration was determined using an Iodine (^125^I) Insulin Radioimmunoassay (RIA) Kit and Iodine (^125^I) Glucagon Radioimmunoassay (RIA) Kit (Beijing North Institute of Biological Technology, Beijing, China) with Gamma Radioimmunoassay Counter (SN-6105, Hesuo Rihuan Photoelectric Instrument Co., Ltd, Shanghai, China). All of the procedures were performed according to the manufacturer’s instructions.

### Fatty acid analysis via gas chromatography

The VFA concentration in the ruminal fluid was determined via gas chromatography (GC) using a FFAP 123–3233, 30-m × 0.32-mm × 0.5-μm, capillary column (Agilent J&W GC Columns, Netherlands) on an Agilent 7890A (Agilent Technologies, USA) as described before with some modifications [16]. Crotonate was used as the internal standard.

The total lipids were extracted from the ruminal fluid and plasma using a mixture of polar and non-polar solvents according to Folch *et al.* at room temperature [17]. The fatty acid methyl esters (FAMEs) were prepared via esterification using sodium methoxide, followed by 14% borontrifluoride in methanol [18]. Heptadecanoic acid methyl ester served as the internal standard and was added to the samples prior to extraction and methylation. The FAME extracts were used for the gas chromatographic analysis of the total fatty acids. The fatty acid composition was determined using GC with a CP 7489, 100-m × 0.25-mm × 0.25-μm, capillary column (Agilent J&W Advanced Capillary GC Columns, Netherlands) on an Agilent 7890A (Agilent Technologies, USA) with an autosampler, flame ionization detector and split injection. The temperature programming was optimal for the separation of the majority of the C18:1 *trans* isomers. The initial oven temperature was 150°C, held for 5 min, then increased to 200°C at a rate of 2°C/min, held for 10 min, then increased to 220°C at 5°C/min and held for 35 min. Helium was used as carrier gas at a flow rate of 1 mL/min. The injector was set at 260°C and the detector at 280°C. The FAMEs were identified by comparing with the retention times of the standard.

### RNA extraction, cDNA synthesis and quantitative real time PCR

The total RNA was extracted from 50 mg of liver tissue using the RNA iso Plus^TM^ reagent (Takara Co., Otsu, Japan) via homogenization on ice. The purity and concentration of the RNA were measured using an Eppendorf BioPhotometer Plus (Eppendorf AG, Hamburg, Germany). The first-strand cDNA was synthesized using 250 ng of the total RNA template using the PrimeScript RT Master Mix Perfect Real Time kit (Takara Co., Otsu, Japan). The primers were designed using Premier 6.0 (Premier Biosoft International, USA) and were based on known cattle sequences or those cited in the published literature [19-21] (Table 2), and the primer efficiencies were evaluated prior to use. The qPCR was performed using the SYBR Premix Ex Taq^TM^kit (Takara Co., Otsu, Japan) on an ABI 7300 Real-Time PCR System (Applied Biosystems, Foster City, CA, USA) according to the recommendations in the instruction manual. The standard PCR protocol was described in the manual: denaturing at 95°C for 15 s, then 40 cycles at 95°C for 5 s, and 60°C for 31 s. Glyceraldehyde phosphate dehydrogenase (GAPDH) served as the housekeeping gene for normalization, and the 2^-ΔΔCt^ method was used for the relative quantification.

**Table 2:** **The gene name, GeneBank accession number, sequence and product size of the primers used for the qRT-PCR**

**Gene**	**Accession #** ^**1**^	**Forward Primer (5’-3’)**	**Reverse Primer (5’-3’)**	**Product Size**
GAPDH^*§*^	NM_001034034	GGGTCATCATCTCTGCACCT	GGTCATAAGTCCCTCCACGA	176
ACC-α^*§*^	NM_174224	AGCTGAATTTTCGCAGCAAT	GGTTTTCTCCCCAGGAAAAG	117
FASN^*§*^	AF285607	GCATCGCTGGCTACTCCTAC	GTGTAGGCCATCACGAAGGT	136
LPL^*§*^	NM_001075120	GGGTTTTGAGCAAGGGTACA	GCCACAATGACCTTTCCAGT	193
FABP1&	FJ415874.1	GTTCATCATCACCGCTGGCT	CCACTGCCTTGATCTTCTCCC	101
PLIN2&	NM_173980.2	TTTATGGCCTCATGCTTTTGC	CTCAGAGCAGACCCCAATTCA	100
ACOX1&	NM_001035289.3	ACCCAGACTTCCAGCATGAGA	TTCCTCATCTTCTGCACCATGA	100
CPT1α&	FJ415874.1	TCGCGATGGACTTGCTGTATA	CGGTCCAGTTTGCGTCTGTA	100
SCD^*§*^	NM_173959.4	TTATTCCGTTATGCCCTTGG	GGTAGTTGTGGAAGCCCTCA	151
DGAT1&	FJ415874.1	CCACTGGGACCTGAGGTGTC	GCATCACCACACACCAATTCA	101
DGAT2&	FJ415874.1	CATGTACACATTCTGCACCGATT	TGACCTCCTGCCACCTTTCT	100
SREBF1c	FJ415874.1	CACTCGTCTTCCTCTGTCTC	GAGTGACTGGTTCTCCATAG	243
SREBF2&	NM_001205600.1	AGAGCAAACTCCTGAAGGGC	GGAGGCGACATCAGAAGGAC	103
SCAP	NM_001101889.1	CATCAAGCTCTACTCCATCC	CAATGGCAGCGTTGTCCAGCA	206
LXRα&	NM_001014861.1	CCCCATGACCGACTGATGTT	TGTCCTTCATCTGGCTCCACC	241
PPARα&	FJ415874.1	CATAACGCGATTCGTTTTGGA	CGCGGTTTCGGAATCTTCT	102

^*§*^published in [19,20]. &published in [21].

^1^Entrez Gene, National Center for Biotechnology Information (NCBI).

### Western blotting analysis

The liver samples were homogenized in RIPA lysis buffer (Beyotime, Shanghai, China) using 0.1 M PMSF using a Dounce homogenizer, and the lysate was centrifuged at 15,000 × g at 4°C for 20 min. The protein concentration of the supernatant was determined using bicinchoninic acid (BCA) and bovine serum albumin as standards (Pierce, Rockford, IL, USA). Equal amounts of protein were separated using 10% SDS-polyacrylamide gel electrophoresis (PAGE) and transferred onto a nitrocellulose membrane (Millipore, Danvers, MA) at 4°C. After blocking with 10% nonfat dry milk in tris-buffered saline at 4°C overnight, the membrane was washed and incubated with a primary antibody directed against SCD (Polyclonal antibodies raised in goat;sc-23016,Santa Cruz Biotechnology, diluted to 1:200) and HRP affinipure rabbit anti-goat IgG as the secondary antibody (E030130-01, Earth Ox, CA, diluted to 1:10,000). Visualization of the SCD protein was performed using the ECL western blot detection system (ECL plus, Beyotime, Shanghai China). The same membrane was then stripped with striping buffer (AR0153, Boster, Wuhan, China)and was normalized against β-tubulin (Polyclonal antibodies raised in goat,sc-9935, Santa Cruz Biotechnology, diluted to 1:200). The procedures for the secondary antibody and visualization were the same as that used for SCD. The ECL signals were recorded using an imaging system (LAS4000, USA) and analyzed using Quantity One (Bio-Rad, USA).

### Statistical analysis

All of the data were expressed as the mean ± SEM. The statistical data analysis was conducted via unpaired or paired Student’s t-tests using IBM SPSS 20.0 Statistics for mac (IBM Inc., New York, USA). A difference was considered to be significant when *p* < 0.05.

## Results

### Rumen pH, LPS content in the rumen and plasma, and the milk composition

**Figure 1:**
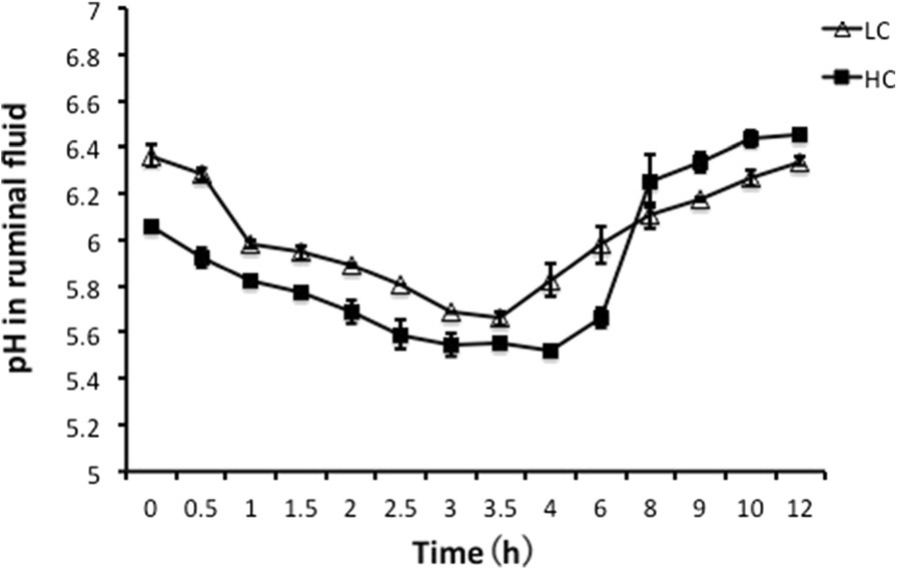
**Comparison of the pH values in the ruminal fluid between the low-concentrate (LC) and high-concentrate (HC) groups.** The data were measured using ruminal fluid samples collected at times ranging from 0 to 12 hours (shown on the x-axis) for three days during the 18^th^ week. Significant differences were observed across all of the sampling times (*p* < 0.05), with the exception of 8 hours after the morning feeding. The error bars indicate the standard error of the mean. The data were compared using Student’s *t*-test.

The pH value in the ruminal fluid is shown in Figure 1. The dynamic pH curve in the HC group was lower than that in the LC group during the long-term experiment. It showed that a pH value under 5.6 lasted for 223 minutes in the HC group, which indicated that SARA was successfully induced. The pH value of the HC group was significantly lower than that of the LC group after the morning feeding for 8 h (*p* < 0.05).

The LPS concentration in the ruminal fluid of the HC group was significantly increased, from 47.17 × 10^3^ EU/mL to 79.04 × 10^3^ EU/mL, compared to the LC group (*p* < 0.01). In the peripheral plasma, the LPS level in the HC group was 0.86 EU/mL, while it was 0.47 EU/mL in the LC group (*p* < 0.001).

Milk samples were collected from the 1^st^ week to the 18^th^ week to determine the change in the milk composition. The results showed that the milk yield, the percentage of milk fat and the milk fat yield decreased significantly in the HC group compared to the LC group (*p* < 0.01). However, the percentage of milk protein increased remarkably in the HC group (*p* < 0.05; Table 3).

**Table 3:** **The LPS, milk yield and milk composition in the dairy cows fed the low-concentrate (LC) and high-concentrate (HC) diets**

**Item**	**Treatment**			
	**LC**	**HC**	**SEM** ^**1**^	**p-Value** ^**2**^
Rumen LPS, EU/mL^1^ (×10^3^)	47.17	79.04^*b*^	7.94	<0.01
Plasma LPS, EU/mL^2^	0.47	0.86^*ab*^	0.08	<0.001
**Milk** ^3^				
Milk yield, kg	28.05	26.92^*b*^	0.30	<0.01
Fat, %	3.40	3.09^*b*^	0.03	<0.01
Fat yield, kg/d	0.95	0.84^*b*^	0.02	<0.01
Protein, %	3.04	3.16^*a*^	0.03	<0.05
Protein yield, kg/d	0.85	0.85	0.02	0.98

^1^SEM = Standard error of the mean between the two groups.

^2^The LPS data were compared using Student’s *t*-test. The milk samples were compared using a paired *t*-test.

^3^The milk samples were obtained from the 1^st^ week to the 18^th^ week.

^*ab*^indicates *p* < 0.001; ^*a*^indicates *p* < 0.05; ^*b*^indicates *p* < 0.01.

### The VFA profile in the ruminal fluid and the plasma biochemical parameters

The VFA profiles in the ruminal fluid and the biochemical parameters in the peripheral plasma between the LC and HC groups are presented in Table 4. When compared to the LC group, the total VFA and lactic acid concentration in the rumen was significantly elevated in the HC group (105.95 *vs.* 92.91, *p* < 0.05 and 1.55 *vs.* 0.96 mmol/L, *p* < 0.01, respectively). The molar proportion (mmol/mol) of propionate was increased in the dairy cows fed the high-concentrate diet (281.83 *vs.* 247.68, *p* < 0.01), but the other proportional concentrations of the individual VFAs were unchanged. The ratio of propionate to butyrate (1.18 *vs.* 1.44, *p* < 0.05) was significantly increased, while the ratio of acetate to propionate (1.84 *vs.* 1.57, *p* < 0.05) was significantly reduced.

**Table 4:** **The ruminal fluid composition, blood metabolites and hormone level in the dairy cows fed the low-concentrate (LC) diet and high-concentrate (HC) diet**

	**Treatment**			
**VFA profile** ^1^	**LC**	**HC**	**SEM** ^2^	**p-value** ^3^
Total VFA(mmol/L)	92.91	107.64^*a*^	3.66	<0.05
Molar proportion, mmol/mol				
Acetate	454.51	440.55	3.10	0.47
Propionate	247.68	281.83^*b*^	2.41	<0.01
Isobutyrate	25.19	24.68	0.38	0.83
Butyrate	210.11	195.99	1.77	0.17
Isovalerate	36.31	34.52	0.67	0.67
Valerate	21.25	19.93	0.24	0.38
Caproate	4.95	2.49^*b*^	0.17	<0.01
Acetate:Propionate	1.84	1.57^*a*^	0.02	<0.05
Propionate:Butyrate	1.18	1.44^*b*^	0.05	<0.01
Lactic acid	0.96	1.55^*b*^	0.20	<0.01
**Plasma biochemical parameter** ^4^				
TG (mmol/L)	0.28	0.21^*a*^	0.05	<0.05
NEFA (mmol/L)	1.16	0.48^*ab*^	0.07	<0.001
TCH (mmol/L)	2.21	1.69^*b*^	0.31	<0.01
GLU (mg/dL)	45.23	56.02^*b*^	4.54	<0.01
**Hormone level** ^4^				
Insulin (μIU/mL)	16.95	21.57^a^	1.01	<0.05
Glucagon (pg/mL)	191.23	161.02	18.28	0.28
Insulin:Glucacon	0.10	0.16^c^	0.01	0.07

^1^The volatile fatty acid and lactic acid concentrations and the mean proportion across the sampling times during the 18^th^ week.

^2^SEM = Standard error of the mean between the two treatments.

^3^The data were compared using a paired *t*-test.

^4^The mean metabolite concentration in the jugular plasma across the sampling times. TG, triglyceride; NEFA: non-esterified fatty acid; TCH, total cholesterol; GLU, glucose;

^*a*^indicates p < 0.05; ^*b*^indicates p < 0.01; ^*ab*^indicates <0.001.

When compared to the LC group, the triacylglycerol (*p* < 0.05), NEFA (*p* < 0.01) and total cholesterol (*p* < 0.01) concentrations were significantly decreased in the peripheral plasma of the HC group. However, the glucose and insulin concentration were significantly enhanced (*p* < 0.01, *p* < 0.05 respectively) in the plasma of the high-concentrate diet group.

### The long-chain fatty acid profiles in the ruminal fluid and the hepatic vein plasma

The LCFA profiles in the ruminal fluid and the hepatic vein plasma are shown in Tables 5 and 6, respectively. The LCFA concentration in the ruminal fluid and the hepatic vein of the HC group was lower than that in the LC group, specifically for palmitate C16:0 (*p* < 0.05) and palmitoleate C18:0 (*p* < 0.05). The desaturation index that was determined by calculating the plasma C16:1n-9/C16:0 ratio was decreased in the HC group (*p* = 0.087). Additionally, a decrease in the Δ^9^ monounsaturated oleic acid (C18:1n-9) concentration was observed in the HC group. Compared with the LC group, the concentration of *cis9,trans 11* CLA was similar in the both rumen and plasma. The concentration of *trans11* C18:1 was increased in rumen of the HC group (*p* = 0.067), while in the plasma, the *trans11* C18:1 content was similar. In regards to α-linolenic acid (C18:3n-3), its content in the HC group was four-fold lower than that of the LC group. However, the content of both C22:0 and C22:1n-9 was significantly increased (*p* < 0.05) in the HC group. The desaturation index of C18:1n-9/C18:0 and *cis9,trans11* C18:2n/*trans11* C18:1n was decreased in the HC group, but no statistical significance was observed. Meanwhile, the presence of longer-chain saturated FAs (C20:0, *p* < 0.01; C21:0, *p* < 0.01), which are produced via ruminal microbial biohydrogenation, was decreased in the HC group.

**Table 5:** **The fatty acid composition in the ruminal fluid of the dairy cows**

**μg/mL**	**Treatment**			
	**LC**	**HC**	**SEM** ^**1**^	**p-Value** ^**2**^
C12:0	2.67	2.05	0.27	0.28
C13:0	3.31	2.92	0.30	0.56
C14:0	254.80	74.26	55.68	0.11
C15:0	6.25	3.47	1.05	0.21
C16:0	147.09	96.84^*b*^	10.48	<0.01
*cis9*C16:1	3.17	2.32	0.11	0.27
C18:0	473.48	281.65^*ab*^	38.02	<0.001
*trans11*C18:1n	4.53	11.40^*c*^	1.93	0.07
C18:1n-9	11.36	8.93	0.65	0.05
C18:2n-6	10.15	7.18^*c*^	0.89	0.09
C20:0	6.28	4.40^*b*^	0.40	<0.01
6C18:3n	0.55	0.75	0.09	0.27
C20:1	0.52	0.35^*b*^	0.04	<0.01
C18:3n-3	2.65	2.04	0.27	0.30
*cis9, trans11*C18:2n	6.61	5.27	0.45	0.15
C21:0	0.76	0.53^*b*^	0.05	<0.01
C22:0	3.53	3.10	0.17	0.23
C23:0	1.21	1.12	0.06	0.51
C24:0	4.26	3.83	0.19	0.29
C24:1	0.69	0.60	0.04	0.29
C22:6n-3	2.21	1.83	0.13	0.17

^1^SEM = Standard error of the mean between the two treatments.

^2^The data were compared using Student’s *t*-test.

^*a*^indicates *p* < 0.05; ^*b*^indicates *p* < 0.01; ^*ab*^indicates *p* < 0.001; ^*c*^indicates 0.05 < *p* < 0.1.

**Table 6:** **The fatty acid composition in the hepatic vein plasma of the dairy cows**

**μg /mL**	**Treatment**			
	**LC**	**HC**	**SEM** ^**1**^	**P-Value** ^**2**^
C12:0	3.57	4.04	0.20	0.26
C13:0	5.92	6.99	0.50	0.32
C14:0	66.06	74.32	13.66	0.79
C14:1	6.47	4.39^*c*^	0.57	0.06
C15:0	8.81	6.43	0.84	0.17
C15:1	1.39	1.25	0.09	0.49
C16:0	92.47	71.10^*a*^	5.14	<0.05
*cis9*C16:1	5.32	2.84^*c*^	0.64	0.05
C18:0	121.33	85.5^*a*^	8.81	<0.05
*trans11*C18:1n	2.92	2.93	0.09	0.96
C18:1n-9	62.24	40.28^*a*^	5.69	<0.05
C18:2n-6	277.99	71.13^*a*^	38.72	<0.05
C20:0	0.89	1.02^*a*^	0.03	<0.05
C20:1	0.45	0.61^*c*^	0.05	0.10
C18:3n-3	25.86	6.6^*b*^	3.74	<0.01
*cis9, trans11*C18:2n	4.56	4.57	0.07	0.98
C21:0	1.27	1.00	0.19	0.51
C22:0	0.97	1.31^*a*^	0.08	<0.05
C20:3n-6	27.01	10.80^*c*^	4.41	0.07
C22:1n-9	1.24	1.57^*a*^	0.08	<0.05
C20:4n-6	33.46	16.13^*b*^	3.84	<0.01
C22:6n-3	10.57	6.20^*ab*^	0.83	<0.001
**Desaturation index**				
cis9 C16:1/C16:0	0.05	0.04^*c*^	0.01	0.09
cis9 C18:1/C18:0	0.51	0.47	0.02	0.37
cis9,trans11 C18:2/tran11 C18:1	1.66	1.55	0.06	0.37

^1^SEM = Standard error of the mean between the two treatments.

^2^The data were compared using Student’s *t*-test.

^*a*^indicates *p* < 0.05; ^*b*^indicates *p* < 0.01; ^*ab*^indicates *p* < 0.001; ^*c*^indicates 0.05 < *p* < 0.1.

### mRNA expression of the genes involved in lipid metabolism in the liver

**Figure 2:**
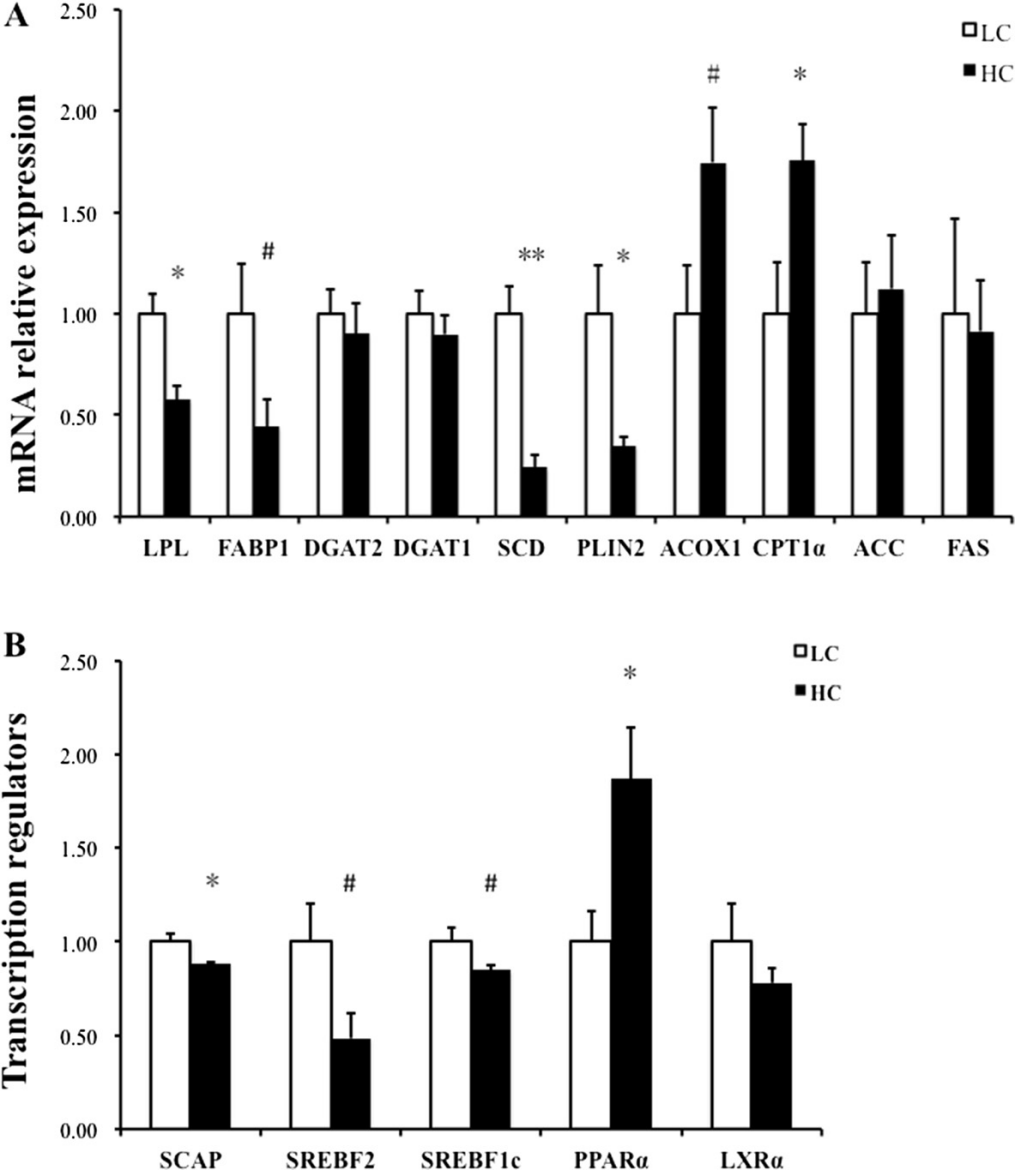
**The hepatic gene expression profile analyzed via real-time PCR.** Each value was normalized to the expression of GAPDH, and data were compared using Student’s *t*-test between LC (n = 4) and HC (n = 4). **A**. The genes involved in desaturation, lipogenesis, fatty acid oxidation, TG synthesis and lipid droplet formation were measured in the liver tissue. The error bars indicate the standard error of the mean. * indicates *p* < 0.05; ** indicates *p* < 0.01; # indicates significance values between 0.05 < *p* < 0.1. **B**. The genes involved in transcriptional regulation were measured in the liver tissue. The error bars indicate the standard error of the mean. * indicates *p* < 0.05; ** indicates *p* < 0.01; # indicates significance values between 0.05 < *p* < 0.1.

The liver mRNA expression levels of the genes involved in lipid metabolism are presented in Figure 2. The expression levels of the genes associated with fatty acid uptake/transport, lipid formation, fatty acid oxidation and transcriptional regulators of lipogenic enzymes were remarkably altered between the HC and LC groups. There was a decrease in fatty acid binding protein 1 (FABP1) expression (*p* = 0.09) in the HC group compared to the LC group, and LPL was significantly down-regulated in the HC group. Compared with the LC group, the expression of perilipin 2 (PLIN2) (*p* < 0.05) was significantly decreased in the HC group, and there was 2-fold down regulation of SCD1 expression in the HC group. The expression of diacylglycerol acyltransferase (DGAT1 and DGAT2) was similar between the two groups, and the expression of ACCα and FAS, which are involved in *de novo* fatty acid synthesis, showed no significant difference between the HC and LC groups. However, the expression of carnitine palmitoyltransferase 1α (CPT1α) was up-regulated in the HC group (*p* < 0.05), whereas the expression of acyl-CoA oxidase 1 (ACOX1) was increased in the HC group (*p* = 0.10).

With respect to transcriptional regulators, the mRNA level of SCAP was down-regulated in the HC group (*p* < 0.05). Meanwhile, both the SREBF1c (*p* = 0.09) and SREBF2 (*p* = 0.08) mRNA expression levels were decreased in the HC group. However, the mRNA expression of PPARα was significantly increased in the HC group (*p* < 0.05). The expression of liver X receptor α (LXRα) showed no significant difference.

### The protein expression of SCD1 in the liver

**Figure 3:**
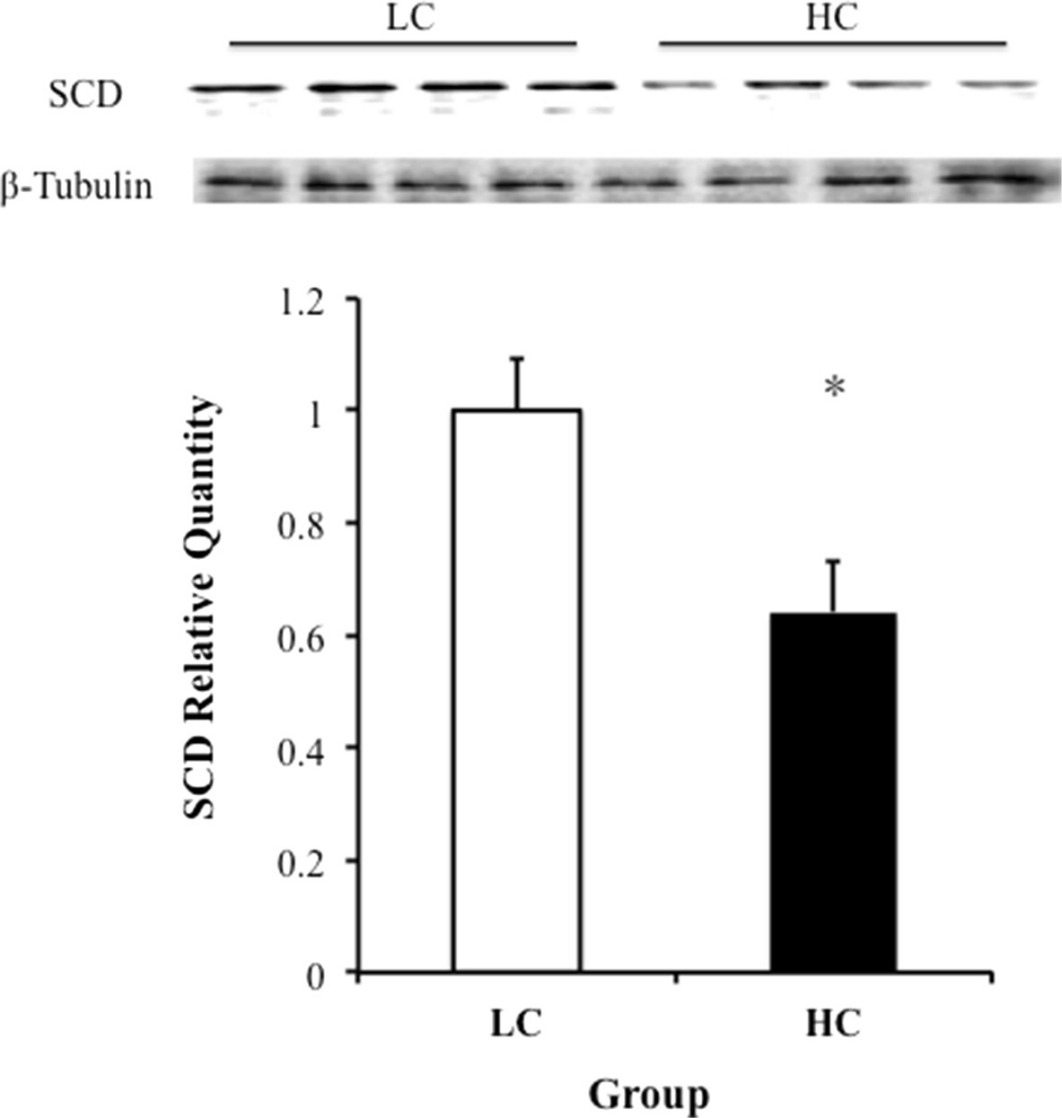
**The western blotting analysis of SCD in the liver.** The SCD content was assessed via western blotting of the livers of the LC (n = 4) and HC (n = 4) cows. The protein was quantified via band density measurements of the western blot. The band densities were normalized to the β-tubulin content within each sample. The data are expressed as the relative amounts of the two groups. * indicates *p* <0.05.The data were compared using Student’s *t*-test.

The protein expression of SCD in the liver is shown in Figure 3. The results demonstrated that the expression of SCD in the liver was significantly down-regulated in the HC group compared to the LC group (*p* < 0.05).

## Discussion

In this study, we showed that an altered fatty acid composition is induced by a HC diet. The reduced oleate and palmitoleate content may be associated with the down-regulated expression of SCD1 in the liver, which primarily resulted from the release of LPS during long-term HC feeding. These findings provide insights into the role of endogenous LPS on hepatic SCD1 expression in dairy cows and its relationship with fatty acid composition.

Previous studies have reported that SARA is characterized by declined feed intake, inflammation and depressed milk fat [12]. In our experiment, the duration of a rumen pH less than 5.6 lasted for 223 min/day in the cows fed a high-concentrate diet, meanwhile, a decrease in the milk yield (kg/d), milk fat (%) and milk fat yield (kg/d) was observed in the HC group. Therefore, our results are consistent with other studies.

Our data demonstrated that the total VFA and lactic acid levels in the ruminal fluid were significantly increased in the HC group. The ratio of acetate to propionate was decreased in the HC group due to an increase in propionate. Early experiments have also presented a low ratio of acetate to propionate in dairy cows fed with easily fermentable carbohydrates [22]. A previous study showed an increased ratio of ruminal propionate to butyrate in repartitioned milk from fat to lactose and protein [23]. In our study, the increased proportion of propionate may be related to glycogenesis [24,25]. Because most volatile fatty acids emerge in the portal vein after absorption from the digestive tract [26], alterations in volatile fatty acid concentrations may influence the metabolism in the liver.

When gram-negative bacteria in the rumen are lysed at low pH values, LPS is released and translocated into the bloodstream, initiating an inflammatory response. In our experiment, the high endogenous LPS content may have triggered metabolic disorders in the digestive tract and liver. It has been documented that the liver has a strong ability to clear LPS [27-29]. A recent study showed that the LPS gene expression profile was altered in the liver of lactating goats fed a long-term high-concentrate diet, and the overall metabolism was shifted towards energy supply, in order to meet the higher energy expenditure demands for tissue anti-inflammation [30].

Our results indicated that SARA also influences plasma metabolites. Cholesterol in the plasma is negatively associated with the presence of LPS in the rumen [31,32], which explains our results of lower cholesterol concentrations in the plasma of the HC group. Among the hormones in peripheral plasma, insulin plays a crucial role in lipid metabolism, particularly in case of feeding cows with a high-concentrate diet. Decreased milk fat yield caused by SARA might have been because of an increased plasma insulin concentration and the ratio of insulin to glucagon [31,33,34], which has shown that high-concentrate diet resulted in greater plasma insulin concentration in our experiment. Due to the higher ruminal propionate and plasma glucose, increased plasma insulin might promote energy expenditure in hepatic through lipolysis and glycolysis, rather than fatty acid synthesis and gluconeogenesis [35], which in turn repressed the expression of lipogenic enzymes, such as SCD1. NEFAs are primarily mobilized from stored TGs in the adipose tissue [36]. In our experiment, the increased propionate and glucose resulted in a decrease in the NEFA concentration in the plasma, which is attributed to their inhibitory effect on adipose tissue lipolysis [35,37,38]. A lower TG concentration was observed in the HC group, which could be explained by the reduced adipose lipolysis and the restriction of biohydrogenation at low rumen pHs [39]. The restricted biohydrogenation led to a decreased saturated FA content, particularly of C16:0 and C18:0, in the rumen.

The fatty acid profiles in the ruminal fluid and the hepatic vein plasma were altered in this study. Decreased saturated FA concentrations in the ruminal fluid reflects the inhibition of biohydrogenation at lower pH values in the rumen of cows fed a long-term high-concentrate diet.

A previous study demonstrated that the conversion of long-chain fatty acids to TGs and phospholipids (PLs) in the livers of dairy cows is dependent on adipose tissue lipolysis [40]. The decrease in the palmitic (C16:0), stearic (C18:0) and oleic (C18:1) content in the liver may be explained by the decreased NEFA levels released from the adipose tissue and the lower production in the rumen. Similarly, a decrease in C18:2n-6 is associated with decreased lipolysis in the adipose tissue [41]. A decrease of both C18:2n-6 and C20:4n-6 in the hepatic vein plasma was observed in the HC group. It was reported that arachidonic acid (C20:4n-6),which comes from the cell cytoplasm, could be synthesized by Δ^5^desaturase from C20:3n-6, and the latter could be desaturated and elongated from C18:2n-6 in the endoplasmic reticulum [42]. In addition, α-linoleic acid (C18:3n-3) could be desaturated and elongated to C22:6n-3, which could explain the decrease in C22:6n-3 in the HC group. Decreased C18:3n-3 may cause an accumulation of TGs [43], due to its function of enhancing the stability of apolipoprotein B:100 [44]. Furthermore, *trans11* C18:1n is considered to bethe precursor of *trans10* C:18:1n, which is a known inhibitor of milk fat in dairy cows [37]. Therefore, its increased levels in the rumen are associated with milk fat depression.

Fatty acid binding protein 1 (FABP1) is related to fatty acid uptake, transport, and metabolism and the activation of PPARα via NEFAs [45]. In our study, the down-regulated expression of FABP1 was likely observed because of the low NEFA concentrations in the peripheral plasma. As increased levels of *trans11* C18:1n in the rumen of the HC group emerged in the portal vein, it may have activated the PPARα pathway, as was demonstrated in a previous in vitro study [46].

In the liver, the activation of carnitine palmitoyltransferase 1α (CPT1α) and acyl-CoA oxidase 1 (ACOX1) is regulated by PPARα [8]. In dairy cows, the above enzymes are responsible for regulating the entry of LCFAs into the mitochondria for oxidation [47,48]. Because of the increased energy demand to resist inflammation during induced SARA, the gene expression of the above enzymes was increased in the HC group, which is consistent with the expression of the transcription factor PPARα. Additionally, shorter-chain fatty acyl-CoA, which is produced via peroxisomal fatty acid β-oxidation, is subsequently channeled to be oxidized completely in the mitochondria [45]. Therefore, the up-regulated expression of CPT1α suggests that hepatic energy export is necessary during induced SARA.

It was reported that the expression of SREBF1c and FAS, which are involved in lipid synthesis, was down-regulated in SCD1 knockout mice [8]. In our study, the expression of the transcription factor SREBF1c was decreased in the HC group, similar to its activation-dependent ligand SCAP, which could further explain the downregulation of SREBF2. In the liver, SREBF1c regulates the genes involved in fatty acid synthesis, while SREBF2 modulates the genes associated with cholesterol biosynthesis [49]. Therefore, the decreased cholesterol in the plasma could be regulated by SREBF2. SCD is a key lipogenic enzyme that regulates the synthesis of monounsaturated fatty acids, particularly oleate (C18:1) and palmitoleate (C16:1) [50]. The transcription of SCD is co-regulated by SREBF1c and PPARα [51,52]. The decreased C16:1 and C18:1n-9 content in the hepatic vein plasma indicated the downregulation of SCD in the dairy cows fed a high-concentrate diet. However, the *cis9,trans11*CLA content was unchanged because the process of *trans11*C18:1n desaturation to *cis9,trans11*CLA via SCD exists in most lipogenic and adipogenic tissues, except in the livers of rats and bovine [53,54]. Moreover, it is considered that the Δ^9^ desaturation index poorly predicts the activity and/or expression of SCD [55]. Similar to SCD, the expression of perilipin 2 (PLIN2), which is involved in the intracellular accumulation of TGs and lipid droplet (LD) formation, was decreased in the HC group [56], which led to the attenuation of LD formation in the liver. Diacylglycerol acyltransferases (DGATs) plays key roles in the synthesis of TGs and very low-density lipoprotein (VLDL) secretion in the liver [57]. In our present study, the similar expression of DGAT1 and DGAT2 between the HC and LC group may be attributed to compensation of the down regulated SCD, which is partly due to a positive correlation between inflammation and fatty liver [58].

A previous study showed that the AMP-activated protein kinase (AMPK) signaling pathway is associated with the inhibition of SCD1 [59]. To some extent, this may contribute to the down regulation of SCD at the mRNA and protein levels. The unchanged mRNA expression of ACCα and FAS may not reflect the phosphorylation status of the enzymes. Therefore, further research is needed to elucidate the underlying mechanism.

## Conclusions

In summary, lipid metabolism in the livers of dairy cows is influenced by long-term high-concentrate diet feeding. Lipopolysaccharide derived from the rumen down-regulates stearoyl-CoA desaturase 1 expression and alters fatty acid composition in the liver of dairy cows fed a high-concentrate diet. Our findings may shed light on the regulation of fatty acid metabolism and reprogramming in the livers of dairy cows fed high-concentrate diets.

## Abbreviations

ACOX1: Acyl-CoA oxidase 1
CPT1: Carnitine palmitoyltransferase 1
DGAT: Diacylglycerol acyltransferase
FABP1: Fatty acid binding protein 1
FAME: Fatty acid methyl esters
LCFA: Long chain fatty acid
LPS: Lipopolysaccharide
LXR: Liver X receptor
PLIN2: Perilipin 2
PPARα: peroxisome proliferator-activated receptor α
SCAP: SREBP cleavage activating protein
SCD: Stearoyl-CoA desaturase
SREBF: Sterol regulatory element binding transcription factor
VFA: Volatile fatty acid
